# All Our Data Will Be Health Data One Day: The Need for Universal Data Protection and Comprehensive Consent

**DOI:** 10.2196/16879

**Published:** 2020-05-28

**Authors:** Christophe Olivier Schneble, Bernice Simone Elger, David Martin Shaw

**Affiliations:** 1 Institute for Biomedical Ethics University of Basel Basel Switzerland

**Keywords:** big data, health data, social media, data protection, guidelines, best practices

## Abstract

Tremendous growth in the types of data that are collected and their interlinkage are enabling more predictions of individuals’ behavior, health status, and diseases. Legislation in many countries treats health-related data as a special sensitive kind of data. Today’s massive linkage of data, however, could transform “nonhealth” data into sensitive health data. In this paper, we argue that the notion of health data should be broadened and should also take into account past and future health data and indirect, inferred, and invisible health data. We also lay out the ethical and legal implications of our model.

## Introduction

### Background

Data intensive software, such as social media, wellness, and mobile health (mHealth) apps, have become ubiquitous in everyday life and are frequently used in a variety of situations. Years ago, social media networks were mostly accessed from traditional computers, but the rising use of smartphones and apps to access those networks has opened a Pandora’s Box regarding data collection, including geolocation, motion data, health-related data, and behavioral data [1]. The collection of additional behavioral data about users was initially very limited, and only a fraction of basic data was collected (eg, IP address, operating system, and browser version). In contrast, current apps on smartphones have begun continuous monitoring of users by harvesting geolocation and motion data, and thus, they have the ability to infer users’ physical and mental health states, for example, to detect signs of depression [2,3] and predict their next likely location [4,5]. Moreover, app companies have collected a tremendous amount of data on individuals’ public and private activities in the digital world, which are being reused not only for the sake of their primary platforms, but also in other lucrative business sectors, such as robotics, life sciences, car manufacturing, and health data provision. Previously, users were able to simply opt out of these services, but this is becoming increasingly challenging nowadays given the monopoly market structure instilled by the companies that drive digital transformation. Indeed, customers are increasingly forced to use these services because they either do not have any other equivalent alternatives in terms of services provided or they are influenced by their peers or parents (in case of children) to use the services. Sometimes these companies nudge users with marketing strategies, such as substantial advantages and discounts offered only on these platforms. This proves to be problematic since to use these now important services, at least to some extent, users have to consent to some mandatory data sharing [6] and consequently expose their privacy.

### Ambiguous Terms and Conditions

In view of the aforementioned facts, it is vital that the relationship between users and companies is transparent and regulated. This relationship is currently mostly defined in the terms and conditions (T&Cs), terms of service, and data privacy notices, which are unfortunately lacking in several aspects in term of enabling potential users to make an informed decision when signing up for a service. For instance, users are not warned about possible harms that might result from their activities on the platforms (eg, linkage of several anonymous data sources could lead to reidentification of otherwise anonymous datasets and lack of awareness of secondary use could undermine user privacy and confidentiality). Additionally, the information provided in the T&Cs of different platforms is not reader-friendly and not succinctly summarized to nudge users to read them thoroughly. They are also not harmonized in the sense that each platform has its own implementation or they are simply not prominent enough during the process of signing up for the service [7,8]. Another weakness of T&Cs is that they do not make it clear that social media and the linkage of several independent unique databases can yield health data. Health data represent a special data category [9], requiring special security and privacy policies for governance. Indeed, many international legislations define health data as special data needing more protection than “usual” nonhealth-related data. The flipside of current legislation is that nonhealth-related data is subject to less strict governance. In the era where a digital phenotype [10] is emerging, data linkage can be very predictive and can be used to, for example, derive personal traits [11], predict psychosomatic diseases [2], and obtain other types of behavioral information.

### Aim of the Paper

This paper presents a new approach for considering data, with the four categories of *direct*, *indirect*, *inferred*, and *invisible health* data, and suggests different types of possible consent frameworks that are up to this challenge, especially as data might not be conceived as distinct health data when produced. We first describe the already recognized categories of direct and indirect health data and then present the other two categories, describe the legal framework in the European Union and United States, and explore the different potential consent mechanisms and their suitability for these four categories of data.

## Ubiquity of Health Data

### Evolution of Health Data

Until the end of the last decade, health data were easily defined, and they included medical records, diagnostic images, laboratory testing data, and data produced by biomedical or clinical means. However, as rightly pointed out by Vayena et al [12], the notion of what is considered health data has considerably evolved. So-called biomedical big data nowadays ranges from data produced by health services, public health activities, and biomedical research to data registering exposure to environmental factors, such as sunlight and pollution, or data revealing lifestyle, socioeconomic conditions, and behavioral patterns, such as those from wellness and fitness apps, social media, and wearable devices. There is thus a paradigm shift from the notion of individual data producers and distinct categories to a more complex notion of a data ecosystem [13].

### Implications of Massively Interlinked Data

The massive amount of data produced and interlinked has an effect on the characterization of individuals today, including their behavioral profile. Jain et al [10] developed the concept of the *digital phenotype*. The digital phenotype, an enlarged notion of the extended phenotype, encompasses the ubiquity of digital technologies and linkage of their data to virtually any other data, possibly resulting in potentially health-relevant data [14]. This notion is underpinned by not only the large numbers of studies conducted by universities that take advantage of the ubiquity of massive amounts of publicly available data, but also research involving the use of apps to predict individual behavior [15].

Further complicating these issues is the possibility that other types of data could be health data one day [16]. For example, some social media data concern exercise, which is highly relevant to health. Most people would agree that exercise data are health-related data and represent an example of indirect health data [10]. However, it is less obvious that other data, such as address and shopping data, location data, smart home data (eg, Amazon Alex and Siri data), smart car data, and articles shared on social media, can be combined with other datasets to infer health data [1,11], and this needs to be made more transparent in the future. It is already possible to use data to infer the degree of exposure to pollution and its likely health effects over long periods, and driving behavior data, such as acceleration patterns, can indicate the risks people take, which again could be used to infer their health conditions when combined with other elements.

### Digitalization of Past Paper-Based Data

At another level, digitalization of past paper-based data could also yield direct or indirect health data (either through digitalization of paper medical records or fitness diaries), and future technologies, such as machine learning, may be able to identify health-relevant uses for data that have not yet even been conceived.

Thus, the days of health data as a distinct category are numbered, and soon, we will have not only direct health data, but also what we term indirect health data, inferred health data, and currently invisible health data (where the relevance to health might be perceptible only by machine intelligence in the future). This new understanding indicates the need for new governance, compliance, and regulatory mechanisms to handle data, protect individuals’ privacy, and uphold the security of such new sensitive data. We briefly examine the current legislation that is relevant to these different categories of health data before continuing our ethical analysis.

## Legal Situation in the United States and European Union

In contrast to the European Union, the United States is not subject to a single overarching data protection law. Data protection issues are implemented at the federal and state level. Table 1 summarizes some of the major federal laws that deal with data protection issues.

**Table 1:** Overview of the most important federal laws on data protection in the United States.

Law	Scope	Main points
Gramm Leach Billey Act	Governs protection of the personal information in the hands of banks, insurances companies, and other companies in the financial service industry.	Addresses “nonpublic information” (NPI) that institutions collect from their customers in connection with the provision of services. Imposes requirements for securing NPI, restricting disclosures, and using NPI. Obligation to notify customers when NPI is improperly exposed.
Fair Credit Reporting Act	Federal law regulating the collection of consumer credit information and access to credit card reports.	Governs how credit bureaus can collect and share information about individual consumers. Businesses check credit reports for many purposes, such as deciding whether to provide a loan or sell insurance to a consumer. This act also gives consumers certain rights, including free access to their own credit reports.
Health Information Portability Act	Protects information held by covered entities concerning health status, provision of health care, or payment for health care.	Breach notification. Data handling by covered entities and definition of safeguards.
Telephone Consumer Protection Act	The order is relevant to any company that uses automated technology to phone or send text messages to consumers.	Regulates telemarketing and forces companies to respect do not call registries.
Family Educational Rights and Privacy Act	Federal law that protects the privacy of student education records.	Offers students the right to correct information about themselves.

In the United States, some privacy frameworks provide a different definition of personal data and the notion of sensitive personal data varies among several federal state laws, with the Californian legislation being the most comprehensive, and among different economic sectors. Health data regulation is mostly included in the Health Insurance Portability and Accountability Act (HIPAA), which only covers entities that are directly related to health care operations, such as health care providers, health plans, and health care clearing houses, and the statement is as follows: “Except as otherwise provided, the standards, requirements, and implementation specifications adopted under this subchapter apply to the following entities: (1) a health plan; (2) a health care clearinghouse; and (3) a health care provider who transmits any health information in electronic form in connection with a transaction covered by this subchapter” (45 CFR § 160.103; 45th Code of Federal Regulations, Office of the Federal Register, United States of America). When data leave these entities, the imposed safeguards by HIPAA do not apply anymore, resulting in less strict regulations. A further guidance issued by the US Food and Drug Administration (FDA) concerns mHealth apps. It addresses, for example, apps that pose a high risk to the public. Apps only fall within its scope if they transform a mobile phone or any other electronic device into a medical device. As the FDA acknowledges in its guidance document, it does not address a substantial number of health data collectors, such as wellbeing apps; websites, especially patient centered portals like PatientsLikeMe; and social networks, and thus, it excludes most indirect, inferred, and invisible health data, which subsequently are subject to the US Federal Trade Commission guidance, resulting in lower safeguards of potentially highly personal data [17].

In contrast, the European Union General Data Protection Regulation (GDPR) offers a comprehensive framework for any kind of personal data and adds different notions to, for example, health or research data. The GDPR treats health data as a special category of data, which is sensitive by its nature, along with several other types, and the statement is as follows: “Processing of personal data revealing racial or ethnic origin, political opinions, religious or philosophical beliefs, or trade union membership, and the processing of genetic data, biometric data for the purpose of uniquely identifying a natural person, data concerning health or data concerning a natural person's sex life or sexual orientation shall be prohibited.” (Article 9, section 1; General Data Protection Regulation, The European Union). Two key points are illustrated by this quotation. First, it is clear that social media data could reveal any of these different sensitive types of data. Second, the term “health data” is not actually mentioned; the phrase used is “data concerning health.” This opens the door to indirect and inferred health data falling within the scope of the GDPR.

Processing of health data is prohibited unless exceptions apply, and one of them is the provision of the individual’s explicit consent. The collection of consent from the data subject remains one of the most common exceptions that organizations processing health data will be able to rely on when it has been explicitly provided and the purpose for processing the data has been explicitly defined.

Comparing the two legislations (EU and US), it can be said that most US regulations on health data remain at the national or state level, such as the HIPAA in the US, which is tightly attached to parts of the health system, such as hospitals, health insurance companies, and pharmacies. Other important data stemming from social networks and other providers are not covered by many of these regulations because they are out of their scope. Furthermore, there are no specific regulations other than broad principles, such as the fair information principles, issued by the US trade commission. In contrast, the GDPR offers at least some boundaries, but there are a number of serious questions on how to interpret the approach of the GDPR to the regulation of social media data. Our broadening of the notion of health data implies both that higher safeguards are applied to nonspecial categories of data and that the lawful grounds for processing such kinds of data need to take care of this situation, and imposing implicit consent could be one approach. As today's consent mechanisms are unable to handle this extra burden, there is an urgent need to foster the development of an alternative consent mechanism. Some examples are delineated later in this article.

The issue of different approaches to data protection has been the subject of disagreement in the literature. Cate et al, who are opponents of a more liberal approach, have argued for self-regulation of data protection principles [18], shifting the burden to users. In view of the recent scandals involving major data intensive companies, this view seems rather inappropriate and neglects the ethical responsibilities of companies toward their customers [19,20].

In contrast, authors like McDermont et al advocate data protection as a human right, with its underpinning ethical principles of privacy, transparency, autonomy, and nondiscrimination. These principles are particularly important in light of the increasing use of large amounts of data and algorithmic prediction [21]. This is also highlighted by Wachter et al, who called for a new right of reasonable inferences to close the accountability gap currently posed by “high-risk inferences,” especially regarding predictions drawn from big data analytics with low verifiability and thus possible damaging effects on individuals [22].

## Extended Notions of Health Data and Consent

Until now, we have assumed that data used in research are primarily generated prospectively (ie, right now or recently) and have excluded data from the more distant past. However, research projects, such as the Time Machine project [23], aim to generate digital copies of vast amounts of past paper-based data. Indeed, digitization of past data is progressing rapidly, for example, in the digital humanities and in the digitalization of large amounts of business and health data [24]. A new notion is thus introduced to the concept of consent. As today’s consent is based on the assumption that the future use of data must be regulated, the linking of old or past data in connection with digitalization would also require consent when an identified or identifiable person is concerned. Consent will therefore have to deal with the past, present, and future use of both past and prospective data.

Consent to use pre-existing data is dealt with in a variety of ways. In medicine, research participants are sometimes invited to give “broad consent” to future reprocessing of data, which is subject to review by a research ethics committee. Such consent is necessarily broad because those giving it will often have no idea of the specific research projects that might use their data in the future. Other models require recontacting participants in order to obtain specific consent for each future project. More radical is the concept of “data donation,” where people grant access to their data under very few limited conditions, if any [25]. However, all of these models are derived from health care and medical research. In the wider context of social media, financial, and location data, consent is based on the initial agreement to the T&Cs as described above and is mostly of a commercial background. Despite the fact that the data could be used in a myriad of ways, even if not ultimately health related, this consent is often entirely uninformed. This is even the case for data that are currently being generated. Awareness and transparency are even lower in terms of possible uses of past paper-based data that are digitalized or future data that might seem irrelevant now but could yield highly relevant health data when combined with other datasets. Current consent for data sharing is to a large extent blind owing to its broad nature. Consent systems actually need to look far back and far forward, as well as in close detail at the present. In essence, consent must be capable of time travel, just as data are capable of time travel.

## Toward Comprehensive Consent in a Hyperconnected World

Several scholars have pointed out that traditional models of obtaining consent have reached their limits in today’s highly data-driven and data-intensive research, and this articulates the need for new forms for obtaining participant consent [26,27]. Concretely, the traditional form of obtaining consent by individually informing participants about their rights and protections is practically impossible in such environments [28-30] owing to the sheer scale and challenges associated with such an endeavor. Several scholars have proposed possible ways to tackle this issue, ranging from information technology-based systems like dynamic consent [31], which offer a better way to inform and maintain a relationship between researchers and participants, to stewardship-based solutions, such as those where a community-based approach assures data governance [32], and radical solutions like data donation [25]. However, most of today’s research projects simply use a digitized version of traditional consent procedures.

As we have already pointed out, past data will become increasingly health relevant. This applies particularly to public and commercial research where it is expected that increasingly more research will be data driven and data will stem from many disparate data sources, including commercial sources. There is thus an urgent need for a better approach of informing customers in a truly informative way. However, as stated above [16], the current approaches are ineffective as T&Cs are too long and written in a too complicated way, undermining what is at the heart of genuinely informed consent, namely the prevention and hold up of basic ethical principles [17,18]. A possible way to tackle this is to introduce harmonized T&Cs, which would need stronger government interventions. Further possibilities are to move toward comic-based consent as developed by Brunschwig for contract law [33] and to implement “nutrition label-like” consent [34] for T&Cs.

The four distinct categories of direct, indirect, inferred, and invisible health data mentioned earlier in this article may each require a differentiated consent solution, and the past, present, and future aspects of consent for the use of big data complicate the situation further.

Direct health data are most easily governed, although they have their own set of challenges. A specific consent system may seem simple but can impose substantial limitations on researchers and prove very burdensome for patients. Broad consent poses its own set of problems, particularly in terms of future data linkage. Even with specific consent to use an individual’s data in a particular project, researchers might also want broad consent to access a participant’s entire medical history and link it to medical records to facilitate follow-up, meaning that consent is provided for health data generated in not only the project itself (the present), but also the years or decades before it (the past) and the many years to come (the future). The growing discussion around data donation illustrates the ethical and legal challenges related to providing consent to ongoing use beyond death for an entire lifetime of genetic and nongenetic health data, some of which may have implications for relatives. In fact, most of the current data protection frameworks, such as GDPR, neglect data donation, and use of data after death is not within their scope (Recital 27 EU GDPR). At least until the point of death, a dynamic consent system seem to be a promising means of controlling different users’ access to past and present medical data and for controlling data linkage with other studies. Another issue complicating the use of direct data concerns mHealth data (ie, data that are gathered when using mHealth solutions). Such apps are mostly overseen by national authorities, and, for instance, in the United States, they need approval from the FDA. Data gathered by those apps need higher protection by law than US regulations and GDPR provide at present. In particular, the issue of consent is rather unregulated, and what could be seen as possible consent (the acceptance of the T&Cs) does not meet the high standards as imposed by traditional consent in research.

Indirect health data, such as exercise, social media, and movement data concerning an individual, are not currently regulated within the health data model in the United States. In the European Union, the GDPR covers such data, but they are not regarded as distinct health data and thus benefit from less protection, as is the case for inferred data. As stated above, consent is usually given via the T&Cs of relevant apps. This must change in the future. Either T&Cs must become much more user friendly and accessible or an entirely different consent model and system more akin to direct health data governance will have to be adopted.

Inferred data are particularly problematic in terms of consent, as they are the result of the combination of two or more datasets of one individual. Consent may have been given to the processing of each of these data points (or sets) but not to their combined processing, which can yield more revealing data not anticipated at the time consent was provided. One way to approach this problem is to make it clear at the point of consent to use direct and indirect health data that their combination with other datasets is a real possibility despite goodwill and efforts on the part of researchers, companies, and other users to prevent it. With further technical developments, it might even be possible to send an alert to a dynamic consent portal for each new instance of combining data points of the individual, enabling tighter and more finely grained control of inferred data. This would ensure that consent is provided when new inferences are made. It is important to bear in mind in the discussion of inferred health data that some data, particularly genetic data, affect not only the data subject but also family members, which is also true for social media data when parents share information of their close relatives and children. Some individuals might be very happy to share all types of data, but this can have ramifications for close relatives, particularly identical twins, in the case of genetic data. In such cases, some form of collective consent may be required.

By its very nature, consent to use invisible health data cannot currently be provided, as we are still blind to the very nature of such data and their potential relevance to health. However, consent can be “future proofed” to a limited extent with careful legislation and regulation. By adopting a similar level of oversight for all types of data concerning a person (the GDPR is a step in this direction), safeguards will be in place once it emerges that seemingly entirely innocuous data can be used by artificial intelligence technologies to yield health findings. Once this transpires, alerts to dynamic consent systems will be a sensible precaution.

As the future use of data cannot be foreseen at present, alert mechanisms play a particularly important role, especially for inferred health data and invisible health data. Given our thesis that any data could turn out to be relevant to health, alerts might well be essential to ensure that people are kept informed when their data are put to a novel use with health implications. Whether citizens would or could have any right to stop the processing of such “new” health data is a difficult question that is outside the scope of this paper, but informing them seems to be a basic ethical requirement. If, as we suggest, all data will become health relevant, it might be impossible or, at least, very impractical for people to stop the processing of all data relevant to their health. Laws, such as the GDPR, will have to keep pace with developments in the conception of health data, as imposing current GDPR standards on all data that might be relevant for health in the future could have great implications for the processing of data, particularly in research. Table 2 summarizes the different categories of health-related data and how consent to use past, present, and future data could be approached.

**Table 2:** Consent based on different health data types related to their temporal origin.

Data type	Past data	Present data	Future data
Direct health data	Specific/broad consent	Direct/specific/broad consent	Direct consent
Indirect health data	Direct consent/terms and conditions	Direct consent/terms and conditions	Direct consent
Inferred health data	N/A^a^	Alerts to dynamic consent systems	Alerts to dynamic consent systems
Invisible health data	N/A	N/A	Alerts to dynamic consent systems

^a^N/A: not applicable.

## Conclusion

With each passing day, billions of gigabytes of direct, indirect, and inferred health data are being recorded, with massive implications for privacy and harm prevention if adequate consent mechanisms for their use are not in place. The possibility of invisible health data complicates the situation further. If all our data will be health data one day, we need to start treating consent to data use with the respect that it deserves. Currently, most data collectors are gaining access to vast amounts of behavioral and health data effectively for free, without having to comply with any safeguards. Broadening the notion of health data, as we have suggested, would cause companies to give more thought to ethical acquisition and processing of data. However, broadening the notion of health data could have an adverse effect on research if it results in excessively burdensome regulations.

Our argument that all data are health data is primarily ethical, but it could have important legal ramifications. In jurisdictions where health data can only be processed with consent, widening the scope of health data in this way would vastly increase the burden on, for example, private companies who process indirect and inferred health data. This might be difficult but ethically appropriate, and the development of more modern and dynamic consent mechanisms could facilitate this shift. Alternatively, legislation could limit the legal scope of health data to direct health data, leaving soft laws and guidelines to regulate other categories of health data.

## Abbreviations

FDA: Food and Drug Administration
GDPR: General Data Protection Regulation
HIPAA: Health Insurance Portability and Accountability Act
mHealth: mobile health
T&Cs: terms and conditions

## References

[1] Ienca M, Ferretti A, Hurst S, Puhan M, Lovis C, Vayena E (2018). Considerations for ethics review of big data health research: A scoping review. PLoS One.

[2] Reece A, Danforth C (2017). Instagram photos reveal predictive markers of depression. EPJ Data Sci.

[3] Saeb S, Zhang M, Karr CJ, Schueller SM, Corden ME, Kording KP, Mohr DC (2015). Mobile Phone Sensor Correlates of Depressive Symptom Severity in Daily-Life Behavior: An Exploratory Study. J Med Internet Res.

[4] Cao H, Lin M (2017). Mining smartphone data for app usage prediction and recommendations: A survey. Pervasive and Mobile Computing.

[5] Do TM, Gatica-Perez D (2014). Where and what: Using smartphones to predict next locations and applications in daily life. Pervasive and Mobile Computing.

[6] Kaplan B (2016). How Should Health Data Be Used?. Camb Q Healthc Ethics.

[7] Cate F, Mayer-Schonberger V (2013). Notice and consent in a world of Big Data. International Data Privacy Law.

[8] (2018). UK Children's Commissioner.

[9] The European Parliament and The Council of The European Union Official Journal of the European Union.

[10] Jain SH, Powers BW, Hawkins JB, Brownstein JS (2015). The digital phenotype. Nat Biotechnol.

[11] Kosinski M, Stillwell D, Graepel T (2013). Private traits and attributes are predictable from digital records of human behavior. Proc Natl Acad Sci U S A.

[12] Vayena E, Blasimme A (2017). Biomedical Big Data: New Models of Control Over Access, Use and Governance. J Bioeth Inq.

[13] Vayena E, Blasimme A (2018). Health Research with Big Data: Time for Systemic Oversight. J Law Med Ethics.

[14] Mayer M, Fernández-Luque L, Leis A (2016). Big Data For Health Through Social Media. Participatory Health Through Social Media.

[15] Harari G, Lane N, Wang R, Crosier B, Campbell A, Gosling S (2016). Using Smartphones to Collect Behavioral Data in Psychological Science: Opportunities, Practical Considerations, and Challenges. Perspect Psychol Sci.

[16] Jain P, Gyanchandani M, Khare N (2016). Big data privacy: a technological perspective and review. J Big Data.

[17] (2016). Federal Trade Commission.

[18] Cate FH, Cullen P, Mayer-Schonberger V (2013). Data Protection Principles for the 21st Century. Maurer Faculty.

[19] Schneble CO, Elger BS, Shaw D (2018). The Cambridge Analytica affair and Internet-mediated research. EMBO Rep.

[20] Schneble CO, Elger BS, Shaw DM (2020). Google's Project Nightingale highlights the necessity of data science ethics review. EMBO Mol Med.

[21] McDermott Y (2017). Conceptualising the right to data protection in an era of Big Data. Big Data & Society.

[22] Wachter S, Mittelstadt B (2019). A Right to Reasonable Inferences: Re-Thinking Data Protection Law in the Age of Big Data and AI. Columbia Bus Law Rev.

[23] Time Machine.

[24] Jones L, Nevell R (2016). Plagued by doubt and viral misinformation: the need for evidence-based use of historical disease images. Lancet Infect Dis.

[25] Shaw DM, Krutzinna J, Floridi L (2019). Defining Data Donation After Death: Metadata, Families, Directives, Guardians and the Route to Big Consent. The Ethics of Medical Data Donation.

[26] Mostert M, Bredenoord A, Biesaart M, van Delden JJ (2016). Big Data in medical research and EU data protection law: challenges to the consent or anonymise approach. Eur J Hum Genet.

[27] Ioannidis J (2013). Informed consent, big data, and the oxymoron of research that is not research. Am J Bioeth.

[28] Christen M, Domingo-Ferrer J, Draganski B, Spranger T, Walter H, Mittelstadt B, Floridi L (2016). On the Compatibility of Big Data Driven Research and Informed Consent: The Example of the Human Brain Project. The Ethics of Biomedical Big Data.

[29] Kaye J, Whitley EA, Lund D, Morrison M, Teare H, Melham K (2015). Dynamic consent: a patient interface for twenty-first century research networks. Eur J Hum Genet.

[30] Jacobs B, Popma J (2019). Medical research, Big Data and the need for privacy by design. Big Data & Society.

[31] Williams H, Spencer K, Sanders C, Lund D, Whitley EA, Kaye J, Dixon WG (2015). Dynamic consent: a possible solution to improve patient confidence and trust in how electronic patient records are used in medical research. JMIR Med Inform.

[32] Blasimme A, Vayena E, Hafen E (2018). Democratizing Health Research Through Data Cooperatives. Philos. Technol.

[33] Brunschwig C (2001). Visualisierung von Rechtsnormen: Legal Design.

[34] Kelley P, Bresee J, Cranor L, Reeder R (2009). A "nutrition label" for privacy. Proceedings of the 5th Symposium on Usable Privacy and Security - SOUPS '09.

